# A Walnut-Enriched Diet Reduces Lipids in Healthy Caucasian Subjects, Independent of Recommended Macronutrient Replacement and Time Point of Consumption: A Prospective, Randomized, Controlled Trial

**DOI:** 10.3390/nu9101097

**Published:** 2017-10-06

**Authors:** Charlotte Bamberger, Andreas Rossmeier, Katharina Lechner, Liya Wu, Elisa Waldmann, Renée G. Stark, Julia Altenhofer, Kerstin Henze, Klaus G. Parhofer

**Affiliations:** 1 Department of Internal Medicine 4, Ludwig-Maximilians University Munich, 81377 Munich, Germany; charlotte.bamberger@med.uni-muenchen.de (C.B.); andreas.rossmeier@med.uni-muenchen.de (A.R.); katharina.lechner@mri.tum.de (K.L.); liya.wu@med.uni-muenchen.de (L.W.); elisa.waldmann@med.uni-muenchen.de (E.W.); julia.altenhofer@med.uni-muenchen.de (J.A.); kerstin.henze@med.uni-muenchen.de (K.H.); 2 Helmholtz Centrum Munich, Institute for Health Economics and Healthcare Management, 85764 Neuherberg, Germany; r.stark@helmholtz-muenchen.de

**Keywords:** walnuts, nuts, lipids, cholesterol, fat, cardiovascular disease, n-3-PUFA, macronutrient replacement, carbohydrate

## Abstract

Studies indicate a positive association between walnut intake and improvements in plasma lipids. We evaluated the effect of an isocaloric replacement of macronutrients with walnuts and the time point of consumption on plasma lipids. We included 194 healthy subjects (134 females, age 63 ± 7 years, BMI 25.1 ± 4.0 kg/m^2^) in a randomized, controlled, prospective, cross-over study. Following a nut-free run-in period, subjects were randomized to two diet phases (8 weeks each). Ninety-six subjects first followed a walnut-enriched diet (43 g walnuts/day) and then switched to a nut-free diet. Ninety-eight subjects followed the diets in reverse order. Subjects were also randomized to either reduce carbohydrates (*n* = 62), fat (*n* = 65), or both (*n* = 67) during the walnut diet, and instructed to consume walnuts either as a meal or as a snack. The walnut diet resulted in a significant reduction in fasting cholesterol (walnut vs. control: −8.5 ± 37.2 vs. −1.1 ± 35.4 mg/dL; *p* = 0.002), non-HDL cholesterol (−10.3 ± 35.5 vs. −1.4 ± 33.1 mg/dL; *p* ≤ 0.001), LDL-cholesterol (−7.4 ± 32.4 vs. −1.7 ± 29.7 mg/dL; *p* = 0.029), triglycerides (−5.0 ± 47.5 vs. 3.7 ± 48.5 mg/dL; *p* = 0.015) and apoB (−6.7 ± 22.4 vs. −0.5 ± 37.7 mg/dL; *p* ≤ 0.001), while HDL-cholesterol and lipoprotein (a) did not change significantly. Neither macronutrient replacement nor time point of consumption significantly affected the effect of walnuts on lipids. Thus, 43 g walnuts/day improved the lipid profile independent of the recommended macronutrient replacement and the time point of consumption.

## 1. Introduction

Although a number of factors are causally linked to cardiovascular disease (CVD), alterations in lipid metabolism, and specifically an elevated concentration of apolipoprotein B (apoB)-containing lipoproteins are considered major risk factors [1]. Therapeutic interventions aiming to reduce LDL cholesterol (LDL-C) levels are of great clinical relevance in the treatment and prevention of CVD. While statins are the cornerstone of drug therapy and should be used in all patients with established CVD [2], lifestyle changes play an important role in primary prevention.

There is strong epidemiologic and clinical evidence that diets rich in omega-3 (n-3) fatty acids are protective and may reduce cardiovascular and overall mortality [3,4,5]. In this context, nuts—especially walnuts—play a key role due to their unique fatty acid composition with high content of unsaturated fatty acids, specifically polyunsaturated fatty acids (PUFA). It has been shown that walnut consumption can affect clinically relevant endpoints (such as cardiac death or endothelial dysfunction), and that this may be mediated through effects on oxidative stress, inflammation, and lipids [6,7,8]. Most of the studies examined the effect of nuts as part of a diet compared with nut-free control diets, which were either low in total fat [9,10] and high in carbohydrate [11], high in fat [12], as part of a Mediterranean diet [13,14], or on a habitual diet [15,16]. Although dietary controls have been variable, the overall results of these clinical trials have consistently shown a cholesterol-lowering effect of regular nut consumption [17].

In a previous study, we investigated the effect of daily consumption of 43 g (1.5 oz) walnuts within a Western-type diet over eight weeks in healthy Caucasian men and postmenopausal women on lipid and glucose metabolism, adipokines, and endothelial function [18]. When compared with a control diet without walnuts, the walnut diet significantly reduced non-HDL cholesterol (non-HDL-C) and apolipoprotein B. Other fasting lipid parameters, as well as biomarkers of endothelial function, postprandial lipids, and glucose metabolism showed no significant changes.

Against this background, the question arises whether it is of any relevance which kind of macronutrient is substituted by the walnuts. Subjects will reduce carbohydrates, fat, or both when they consume walnuts, which may influence the lipid modifying effect. Furthermore, we wanted to investigate whether the time point of consumption (meal or snack) has an effect on the lipid profile, as it has been shown for almonds (which have a more pronounced effect when taken as a snack) [19].

The aim of the current study was therefore to investigate the effect of walnut consumption in subjects who isocalorically replaced fat, carbohydrates, or both with walnuts as a snack or as a meal.

## 2. Subjects and Methods

### 2.1. Subjects

Study subjects were recruited by advertisement in local newspapers and through posters in the outpatient clinic. We included healthy non-smoking subjects older than 50 years (men and postmenopausal women) with LDL-C <190 mg/dL, triglycerides (TG) <350 mg/dL, and a body mass index (BMI) <35 kg/m^2^. We excluded persons with a history of cardiovascular and atherosclerotic disease, a known allergy to tree nuts, a vegan or ovo-lacto vegetarian lifestyle, and patients on regular medication (except stable treatment of thyroid disease and hypertension). A total of 268 subjects were screened. After the initial screening, 204 subjects were randomized and included in the trial. Ten subjects did not complete the study. In total, 194 subjects (60 men and 134 women) completed the study and were included in the main data analysis (complete case analysis). However, an intention to treat analysis was also performed.

### 2.2. Study Design

**Figure 1:**
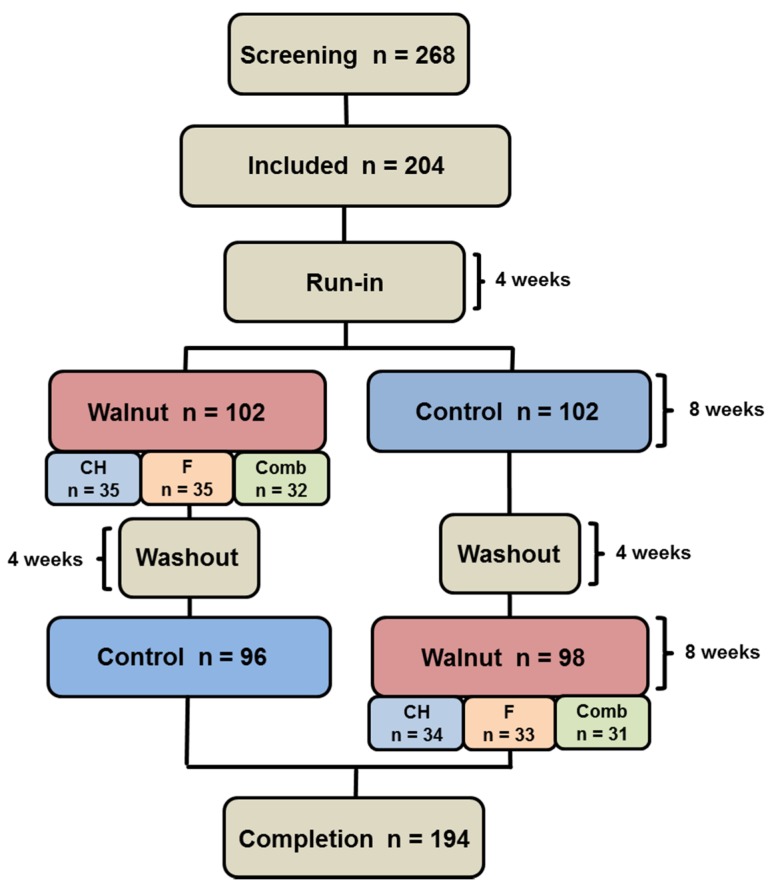
Flowchart of study subjects. In total, 204 subjects were randomized. Ten subjects dropped out due to disease (*n* = 2), medication (*n* = 1), personal reason (*n* = 5), protocol violation (*n* = 2). A total of 194 subjects were included in statistical evaluation. CH: carbohydrate restriction, F: fat restriction, Comb: combined carbohydrate and fat restriction.

The study was designed as a randomized, controlled, prospective, cross-over study. Each subject followed a nut-free Western-type diet during a 4-week run-in period. Thereafter, subjects were randomized to two different diet phases, each lasting for 8 weeks (separated by a 4-week washout period, during which study subjects again followed a nut-free Western-type diet). One group (*n* = 96) first followed a walnut-enriched diet (43 g of shelled walnuts/day) and then switched to a nut-free control diet. The other group (*n* = 98) followed the diets in reverse order (Figure 1).

**Figure 2:**
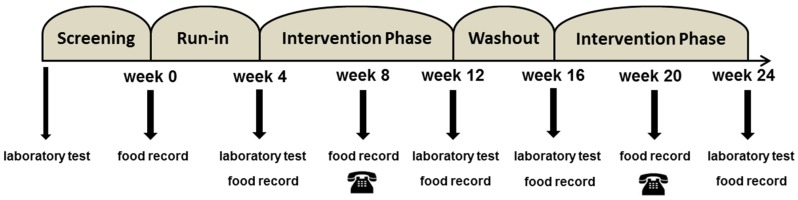
Flowchart of study procedures.

Study duration was six months (24 weeks) for each subject (Figure 2). Before starting the run-in period, subjects underwent counseling by a nutritionist, who evaluated dietary habits using a four-day dietary report that had been prepared by the participants during the preceding days. At the beginning and end of each diet phase, subjects were evaluated in the study center. Subjects met with a nutritionist at each follow-up visit, and a telephone follow-up was conducted regularly once during each diet phase. Prior to each visit, subjects were instructed to keep a four-day food record.

The recommended background diet during the run-in and the washout phases was a nut-free Western-type diet consisting of 50% carbohydrates, 35% fat (15% saturated fat), and 15% protein. Subjects were instructed not to consume dietary supplements containing omega-3 fatty acids (such as fish oil and linseed oil) during the entire study. At the beginning of the walnut diet, each subject was provided with a daily serving of 43 g of shelled, prepackaged walnuts (this corresponds to approximately additional 300 kcal/day mostly as fat). The subjects were randomized into three different diet groups, in which they were advised to reduce the intake of either carbohydrates (CH, *n* = 62), fat (*n* = 65), or both (*n* = 67) during the walnut diet. They were instructed to replace either 70 g carbohydrates or 30 g of (saturated) fat with the walnuts. Subjects assigned to the third group were advised to replace both macronutrients (35 g carbohydrates and 15 g fat) with the daily walnut serving. These recommendations were food based, i.e., on the basis of individual food reports (free text), a nutritionist recommended specific measures to adjust the diet. Furthermore, the groups were randomized to consume walnuts either during a meal or as snack. Study subjects who were randomized to eat walnuts during a meal were instructed to eat unprocessed walnuts with main dishes. As outlined in Figure 2, each individual completed seven food reports to give recommendations and to monitor compliance. Dietary reports were evaluated using PRODI® 6.2 Nutri-Science GmbH (Nutri-Science GmbH, Freiburg, Germany). Consumption of the daily serving of walnuts was controlled by evaluating the food records and by asking study participants. Study participants received detailed study material that helped them assess their individual caloric intake. The same material showed how to replace carbohydrates or fat sources in their daily diet, i.e., by reducing the intake of bread or pasta to save carbohydrates.

### 2.3. Laboratory Measurements

EDTA (Ethylenediaminetetraacetic acid)-containing blood samples were used to analyze fasting lipid parameters. Very low density lipoproteins (VLDL) (*d* < 1.006 g/mL) were separated by ultra-centrifugation (Beckman L-60 centrifuge with 50.4 Ti rotor). Total cholesterol (TC), TG, VLDL-cholesterol (VLDL-C), apoB, lipoprotein (a) (Lp (a)) concentrations, fasting glucose, and C-reactive protein CRP were directly measured on an autoanalyzer (Respons® 910, DiaSys Diagnostic Systems, Holzheim, Germany) by using ready-to-use reagent kits (DiaSys Diagnostic Systems, Holzheim, Germany). HDL cholesterol (HDL-C) was measured after precipitation with heparin and manganese (II) chloride (polyanion precipitation). LDL-C was calculated by subtracting HDL-C from the total cholesterol in the infranatant of the ultracentrifugation. Non-HDL-C was calculated by subtracting HDL-C from TC. Hemoglobin A1c (HbA1c) concentrations were analyzed in the Department of Clinical Chemistry at the University of Munich Medical Center using routine laboratory tests. Endothelial activation markers sVCAM-1 and endothelin-1 were determined using commercially available ELISA kits (Human sVCAM-1/CD106 Quantikine ELISA Kit DVC00; Endothelin-1 Quantikine ELISA Kit DET100; R&D Systems, Minneapolis, MN, USA).

### 2.4. Statistical Analysis

Our primary outcome measure was fasting non-HDL-C. Secondary outcome measures included: fasting TC, TG, VLDL-C, VLDL-TG, LDL-C, HDL-C, apoB, Lp (a), fasting plasma glucose, HbA1c, hsCRP, BMI, and waist circumference. Based on our previous study [18], we estimated an average difference between the control and the walnut diet of 10 mg/dL in the primary endpoint (change in non-HDL-C) in each of the three subgroups. The power calculation indicated that 40 subjects per group were needed to complete the treatment periods to detect the indicated mean difference in non-HDL-C. We also advised subjects to consume walnuts either as snacks or with meals. The power to detect a 20% difference between subjects receiving walnuts as a snack vs. walnut as a meal was 30% for each subgroup. However, if all of the subjects receiving walnuts as snacks are compared to those receiving walnuts as meals (independent of the macronutrient being substituted), then the power increases to >50%. Changes in mean fat, protein, carbohydrate, and calorie consumption in the different dietary periods were examined with a two-tailed unpaired *t*-test or non-parametric Wilcoxon as appropriate. Results were arranged so that our dataset contained one observation for findings in the control phase—including baseline, post and change in the lipid values—and another observation for the findings in the walnut phase with corresponding values for each subject. For the analyses, we used a mixed generalized linear model accounting for these repeated measures and additionally adjusted for the baseline parameter values, age, gender, baseline BMI, and treatment sequence, type of diet reduction (fat, carbohydrate or both) and ingestion of walnuts as a meal or a snack. Comparisons between the effect of walnuts and controls on changes in the parameters of lipid metabolism and glucose metabolism were thus examined with the generalized linear regression models described above, adjusting for the factors described above. The effect of macronutrient replacement and walnuts as meal or snack in the walnut phase on lipid parameters were analyzed using a model that adjusted for baseline parameter value, age, gender, baseline BMI, treatment sequence, type of diet reduction (fat, carbohydrate or both) and ingestion of walnuts as a meal or snack. For the comparison of treatment to the control phase for macronutrient replacement and walnuts as a meal or snack, an interaction term was included in separate regression analyses (either treatment*type of diet reduction or treatment*meal/snack). Statistical significance was set at *p* ≤ 0.05. Primary analyses were performed in 194 subjects completing the study (complete case analyses). In addition, we also performed intention to treat analyses (ITT) of all 204 randomized subjects with all of the missing values imputed using single Markov chain Monte Carlo (MCMC) imputation. ITT analyses studying the effect on fasting non-HDL-C (primary outcome measure) were also performed with five imputation data sets. Randomization (blocking of 12; SAS proc factex) and statistical analysis were performed using SAS 9.3 (SAS Institute, Cary, NC, USA). Data were blinded for laboratory analysis.

### 2.5. Ethics

The study was conducted according to the guidelines in the Declaration of Helsinki and the ICH Harmonized Tripartite Guideline for Good Clinical Practice. The study protocol was approved by the ethics committee of the Faculty of Medicine of the University of Munich. After adequate information, all of the subjects provided written informed consent. The study was registered at ClinicalTrials.gov (NCT02329067) and performed between February 2015 and May 2016 at the University of Munich Medical Center. Walnuts were provided by the California Walnut Commission (Folsom, CA, USA).

## 3. Results

In total, 134 females and 60 males (mean age 63 ± 7 years) completed the trial. Baseline characteristics were measured after the run-in phase, and are shown in Table 1. Baseline characteristics did not differ significantly between groups.

**Figure 3:**
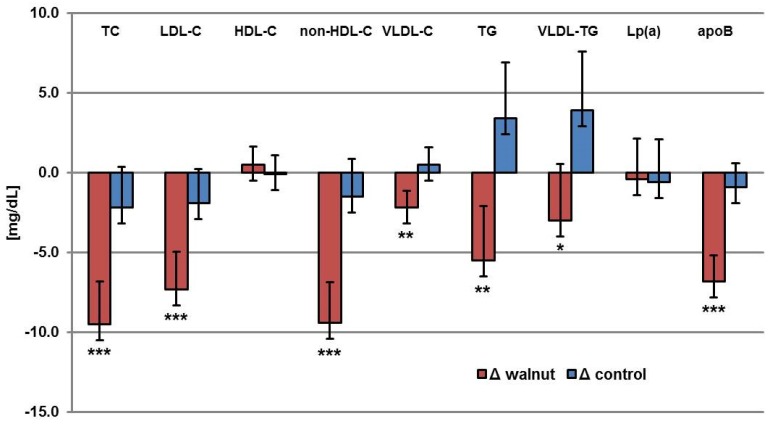
Changes in fasting plasma lipid levels and apoB concentrations from baseline (mg/dL) during the walnut diet phase and control diet phase. (*n* = 194) Values are mean ± SEM. *p*-value refers to differences between walnut phase and control phase; * *p* ≤ 0.05, ** *p* ≤ 0.01, *** *p* ≤ 0.001. TC, total cholesterol; LDL-C, low density lipoprotein-cholesterol; HDL-C, high density lipoprotein-cholesterol; VLDL-C, very low density lipoprotein-cholesterol; TG, triglycerides; Lp (a), lipoprotein (a); apoB, apolipoproteinB.

Walnut consumption significantly reduced non-HDL-C, TC, LDL-C, VLDL-C, TG, VLDL-TG and apoB when compared with the control period (Table 2, Figure 3), but did not significantly change HDL-C and Lp (a). Intention to treat (ITT) analysis with the imputation of five datasets also shows that treatment with walnuts results in a decrease in non-HDL cholesterol, which is −7.61 (STD err: 1.77; *p*-value: <0.0001) lower in the walnut phase than in the control phase. Further ITT analyses are shown in Supplemental Table S1.

**Figure 4:**
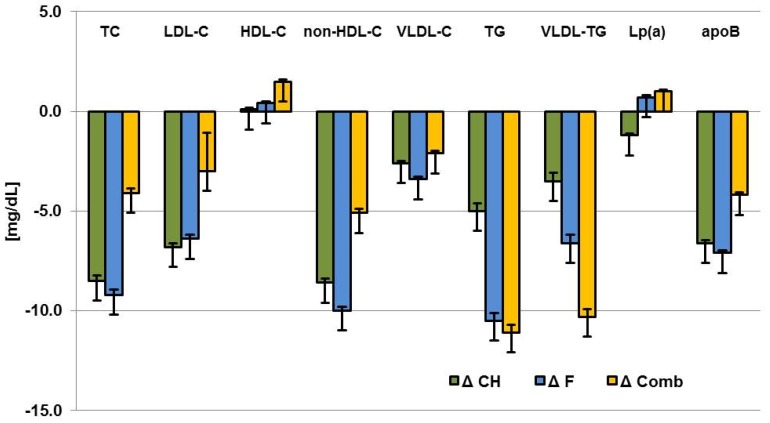
Changes in fasting plasma lipid levels and apoB concentration from baseline in subgroups (mg/dL). Values are mean ± SEM. CH: carbohydrates reduced in walnut-phase (*n* = 62); F: Fat reduced in walnut-phase (*n* = 65); Comb: both fat and carbohydrates reduced in walnut-phase (*n* = 67). TC, total cholesterol; LDL-C, low density lipoprotein-cholesterol; HDL-C, high density lipoprotein-cholesterol; VLDL-C, very low density lipoprotein-cholesterol; TG, triglycerides; Lp (a), lipoprotein (a); apoB, apolipoproteinB.

The effect of walnut consumption on lipid parameters was independent of the macronutrient replaced by walnuts (CH vs. fat vs. combined) (Table 3, Figure 4). ITT analysis with the imputation of five datasets showed that there is no difference in the change in non-HDL cholesterol according to type of diet reduction when looking at the walnut group: Δcarbohydrate vs. Δfat: −0.69 (STD err: 3.45; *p*-value: 0.8419); Δcarbohydrate vs. Δcomb: −3.42 (STD err: 3.35; *p*-value: 0.3072); Δfat vs. Δcomb: −2.73 (STD err: 3.29; *p*-value: 0.4062), and the comparison of walnut to controls (Δcarbohydrate vs. Δfat: −2.69 (STD err: 4.23; *p*-value: 0.5267; Δcarbohydrate vs. Δcomb: −4.67 (STD err: 4.23; *p*-value: 0.2716). Further ITT analyses are shown in Supplemental Table S2.

Similarly, the effect was independent of whether walnuts were consumed as a snack or with a meal (Table 4). ITT analysis with the imputation of five datasets shows that walnuts given as a meal versus a snack results in a change in non-HDL cholesterol, which is −5.15 (STD err: 2.70; *p*-value: 0.0587) and lower when given with meals than as a snack (only looking at the walnut phase). Comparing the results of both phases shows a change in non-HDL cholesterol, which is −2.05 (STD err: 3.51; *p*-value: 0.5595) lower when given with meals than as a snack. Further ITT analyses are shown in Supplemental Table S3.

**Figure 5:**
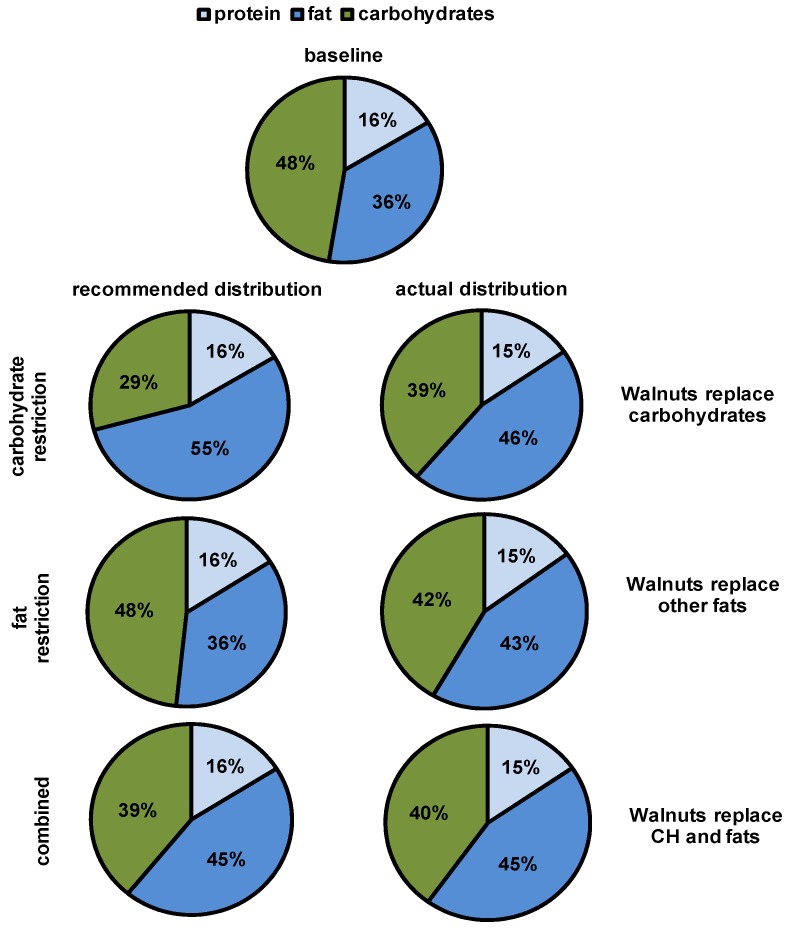
Adherence to recommended macronutrient replacement during walnut consumption. Percentage of daily total calories at baseline, as well as the recommended and the actual distribution of calories, were calculated by analyzing dietary reports.

Analysis of self-reported nutrient intakes from the four-day dietary reports showed that the study subjects did not fully maintain an isocaloric diet as recommended during both intervention periods, but increased caloric intake during walnut consumption (+423 kJ (101 kcal) vs. +88 kJ (21 kcal), *p* = 0.042). In the CH group, the proportion of fat increased significantly (+20.4 g vs. +1.6 g, *p* < 0.001), whereas the amount of carbohydrates decreased significantly (−23.7 g vs. +0.6 g, *p* = 0.004) during walnut consumption. In the fat group, the intake of carbohydrates did not significantly differ between the walnut and control diet (−9.0 g vs. +2.8 g, *p* = 0.067), but fat intake significantly increased during walnut consumption (+21.6 g vs. +7.2 g, *p* < 0.001). In the third group (Comb), a significant increase of fat during the walnut diet could be observed (+21.4 g vs. +0.1 g, *p* < 0.001), while carbohydrate consumption did not change significantly. A detailed analysis of the dietary reports indicates that subjects did not fully comply with the recommended substitution (Figure 5). Thus, the CH group reduced carbohydrates, but did not fully compensate for the additional walnuts. Instead, other fat was also reduced, and overall energy intake increased. Similarly, the fat group did not adequately reduce other fat during walnut consumption, but also reduced carbohydrates and increased overall energy consumption.

When the three walnut diets were compared with each other, no significant difference was detected, which indicated that subjects of all of the diet groups ate a similar diet during the walnut phase, despite different recommendations. A subgroup analysis of those subjects who properly followed the dietary instructions (10–30 subjects in each group) showed no significant difference in the decrease of blood lipids during walnut consumption between the different diet groups.

Walnut consumption did not affect hsCRP, endothelial markers, and fasting glucose, but was associated with a significant increase in HbA1c (+0.07, *p* = 0.0057).

## 4. Discussion

Our study demonstrates that supplementing 43 g of walnuts daily for eight weeks significantly decreases fasting lipid parameters, including non-HDL-C, apoB, TC, LDL-C, VLDL-C, TG, and VLDL-TG in healthy individuals, irrespective of whether the subjects are instructed to replace carbohydrates, fat, or both. We did not observe changes in hsCRP, biomarkers of endothelial dysfunction and anthropometric parameters, including waist circumference and body weight, but observed a minimal increase in HbA1c (no effect on fasting glucose). The observed effects did not change materially after adjustment for gender, age, BMI, and diet sequence. Furthermore, the results were virtually identical when an intention to treat analysis was performed.

### 4.1. Lipid-Lowering Effect

Within the tree nut family, walnuts have been found to be particularly promising in cardioprotective health. As one of the first, Sabaté et al. demonstrated the cholesterol-lowering effect of daily walnut consumption [20]. Our results showed a significant reduction in LDL-C (−7.3 mg/dL), which represents a reduction of 5.0%. These findings resonate with the results of our previous study [18], in which a similar pattern was observed (non-HDL-C: −5.8%, TC: −3.9%, apoB: −6.2%, VLDL-C: −13.2%, TG: −5.4%, VLDL-TG: −4.0%). Our results suggest that the increased n-3-PUFA intake (15.0%) was principally responsible for the cholesterol-lowering effect of walnuts. There is evidence that a high n-3-PUFA intake provides cholesterol-lowering effects through several potential mechanisms [21]; however, the exact underlying mechanisms are still not fully understood.

### 4.2. Macronutrient Replaced and Time Point of Consumption

In our previously published study, we asked subjects to reduce saturated fatty acid intake while eating walnuts, but observed that carbohydrates and fat were reduced. This led us to question whether replacing different macronutrients had a differential effect on the plasma lipid profile. Several lines of evidence indicate that the effect could be different if walnuts replace carbohydrates or fat. The reduction of carbohydrate intake has been shown to decrease plasma TG levels and to increase HDL-C without affecting LDL-C levels [22]. On the other hand, the replacement of saturated fat with monounsaturated fat has been associated with decreased TC, LDL-C, and HDL-C [23]. The replacement of saturated fat with carbohydrates has been reported to result in lower TC, LDL-C, and HDL-C levels, but also a slight increase in TG [24,25]. A replacement of saturated fat with both monounsaturated fat and carbohydrates effectively lowered LDL-C; however, the replacement with monounsaturated fat was associated with lesser reductions in HDL-C and lesser increases in TG concentrations [26]. Replacing saturated fat with polyunsaturated fat and/or monounsaturated fat has been shown to be equally efficacious at reducing lipoprotein levels [27]. Based on these findings, we hypothesized that the greatest lipid-lowering effect would be seen in the group that replaced fat by walnuts. However, there was no statistically significant difference in lipid reduction between the diet groups.

Although we invested considerable time and effort (frequent visits with a nutritionist; detailed analysis and discussion of food protocols), our subjects did not fully comply with the recommended diet (i.e., substitution of carbohydrates or fat or both for walnuts). In fact, there was no statistical difference between the three diets, indicating that subjects had a very similar diet, despite very different recommendations. This probably reflects the “real world”, and indicates that most people will cut down on carbohydrates and fat (non-walnut fat) if they consume walnuts (irrespective of recommendations). To ensure better compliance of the dietary recommendations, a much more controlled setting is needed, such as an in-patient setting or at least a setting where all of the meals are provided. However, this setting would introduce other confounders (e.g., restricted physical activity). On the other hand, it is questionable whether the results would be different, as a subgroup analysis of those subjects who properly followed the dietary instructions also showed no significant difference concerning the effect of walnut consumption on lipids. This indicates that the effect is independent of whether walnuts replace carbohydrates, fat, or both. This supports the notion that walnuts improve the lipid profile, not only independent of the recommendations, but also independent of the actually executed diet.

In our study, the effect of walnut consumption on lipids did not depend on whether walnuts were consumed with meals or as snacks. In a previous study describing the effect of almonds, it has been demonstrated that the beneficial effect on various metabolic parameters was most pronounced when almonds were taken as snacks [19]. It can be assumed that nuts when consumed as a snack alone may result in enhanced acute satiety responses, thus keeping the balance to the overall energy intake [28,29]. On the other hand, almonds have been shown to significantly reduce post-prandial glucose excursion by slowing digestion, in addition to lowering serum cholesterol levels when consumed with a meal [30]. Further trials may be necessary to validate whether the time and form of consumption influence the effect on blood lipid levels.

### 4.3. Other Findings

The secondary parameter hsCRP and the endothelial markers VCAM-1 and ET-1 remained unaffected during the walnut diet. These findings are consistent with the results of our previous study [18], as well as other studies focusing on the effect of a walnut or n-3 PUFA-enriched diet on endothelial and inflammatory markers [7,14].

To our surprise, we observed a slight, clinically irrelevant but significant increase in HbA1c (5.4 ± 0.3 vs. 5.5 ± 0.3). These findings contrast with epidemiological studies that show an improvement in glucose parameters after nut consumption in subjects with or at risk of diabetes [28,31,32,33]. The antidiabetic effects of n-3 PUFAs are expected to result from enhanced insulin sensitivity due to an increased expression of insulin receptors [34]. Several potential explanations can be offered for the observed change in glucose metabolism. First, our study subjects consumed more calories during the walnut phase, which may affect glucose metabolism and HbA1c levels. Second, statin therapy, which dramatically decreases LDL-C, and genetic variants leading to lower LDL-C, both also negatively affect glucose levels [35,36]. Thus, theoretically, the small decrease in LDL-C could be linked to the increase in HbA1c. Third and most likely, it could be a chance finding. Nevertheless, this aspect deserves further investigation.

Body weight and BMI remained stable during both walnut and control diet. These findings are consistent with our prior research, where daily walnut consumption did not lead to a significant change in anthropometric parameters. Indeed, data from both observational and clinical studies show that supplementing nuts to habitual diets does not cause weight gain [37,38], despite the fact that subjects often seem to consume more energy when eating nuts. However, recent data indicate that the metabolizable energy content of walnuts is lower than predicted [39]. According to these data, one 43 g serving of walnuts can be estimated to contain about 942 kJ (225 kcal, thus 5.22 kcal/g metabolizable energy), about 314 kJ (75 kcal) less than previously assumed.

### 4.4. Strengths and Limitations

A major strength of our study is its prospective, randomized, cross-over design with washout periods to ensure good basic conditions. Furthermore, our study set the focus on nutritional replacement to investigate whether the exchange of macronutrients for walnuts and the time point of consumption may have an impact on the lipid-lowering effect of walnuts. On the other hand, the interpretation is limited by the fact that the study relied on self-reported food records completed by the participants. These data may be highly susceptible to recall bias. Furthermore, dietary intake was not monitored daily, but rather recorded for four consecutive days. These were in turn interrupted by non-representative conditions (e.g., public holidays). Finally, in order to standardize baseline diet, patients had to follow a Western-type diet during the run-in period, which by itself represented a dietary change for many subjects.

## 5. Conclusions

Our study demonstrates that supplementing 43 g of walnuts for eight weeks favorably changes plasma lipids by lowering total cholesterol, LDL-C, non-HDL-C, triglycerides, and apoB. Thus, embedding walnuts in a healthy subject’s nutrition may be an effective strategy for reducing the risk for cardiovascular disease. It seems irrelevant which macronutrients are replaced and whether walnuts are consumed in meals or as snacks.

## Figures and Tables

**Table 1:** Baseline characteristics of all study subjects

Demographic and Anthropometric Values (*n* = 194)	Baseline	(Min–Max)
Gender	m = 60 f = 134	
Age (years)	63 ± 0.54	(50–86)
BMI (kg/m^2^)	25.4 ± 0.29	(17.2–35.3)
Waist circumference (cm)	83.5 ± 0.81	(62–108)
**Fasting Metabolic Parameters**		
TC (mg/dL)	231.6 ± 2.7	(119–330)
Non-HDL-C (mg/dL)	161.7 ± 2.6	(71–243)
LDL-C (mg/dL)	146.3 ± 2.3	(54–206)
HDL-C (mg/dL)	68.6 ± 1.2	(35–111)
Triglycerides (mg/dL)	101.2 ± 4.0	(31–296)
Glucose (mg/dL)	91.4 ± 0.7	(74–118)
HbA1c (%)	5.4 ± 0.02	(4.0–6.4)

Values are mean ± SEM and median (range) for lipoprotein (a); TC, total cholesterol; HDL-C, high density lipoprotein-cholesterol; LDL-C, low density lipoprotein-cholesterol.

**Table 2:** Adjusted effect on fasting lipids, apolipoprotein B (apoB) and lipoprotein (a) (Lp (a)) (mg/dL) of intake of walnuts compared with controls in 194 subjects completing the study.

Parameter	Baseline_W_	ΔWalnut	Baseline_C_	ΔControl	p *
TC (mg/dL)	231.7 ± 2.7	−9.5	231.6 ± 2.5	−2.2	0.0003
LDL-C (mg/dL)	146.3 ± 2.3	−7.3	146.0 ± 2.1	−1.9	0.0009
HDL-C (mg/dL)	68.6 ± 1.1	0.5	68.8 ± 1.2	−0.1	0.297
non-HDL-C (mg/dL)	163.1 ± 2.6	−9.4	162.8 ± 2.4	−1.5	<0.0001
VLDL-C (mg/dL)	16.7 ± 1.1	−2.2	16.8 ± 1.1	0.5	0.0021
TG (mg/dL)	101.2 ± 3.4	−5.5	102.8 ± 3.5	3.4	0.0043
VLDL-TG (mg/dL)	74.3 ± 3.6	−3	77.8 ± 3.7	3.9	0.0355
Lp (a) (mg/dL)	12 (1–139)	−0.4	11.5 (2–173)	−0.6	0.8079
apoB (mg/dL)	109.9 ± 1.6	−6.8	109.5 ± 1.5	−0.9	<0.0001

Values are mean ± SEM and median (range) for lipoprotein (a); * *p*-value refers to comparison between ΔWalnut vs ΔControl; change in parameters and *p*-value calculated with multivariate regression adjusting for age, gender, baseline parameter, baseline BMI, treatment sequence, type of diet reduction, and walnuts as a snack or meal. The intention to treat analysis in all 204 subjects randomized showed that the same parameters changed significantly, with only marginally different *p*-values; TC, total cholesterol; LDL-C, low density lipoprotein-cholesterol; HDL-C, high density lipoprotein-cholesterol; VLDL-C, very low density lipoprotein-cholesterol; TG, triglycerides; Lp (a), lipoprotein (a); apoB, apolipoproteinB.

**Table 3:** Effect of walnut consumption on fasting lipids, apoB and Lp (a) when walnuts replace carbohydrates, fat, or both.

	Walnuts				Difference between Walnuts and Control			
Parameter	ΔCarbohydrate	ΔFat	ΔComb	*p*	ΔCarbohydrate	ΔFat	ΔComb	*p*
TC (mg/dL)	−11.9 ± 2.7	−9.7 ± 2.6	−6.2 ± 2.8	0.3158	−8.5 ± 3.5 (0.0148)	−9.2 ± 3.4 (0.0070)	−4.1 ± 3.3 (0.2202)	0.5113
LDL-C (mg/dL)	−9.0 ± 2.3	−6.9 ± 2.3	−5.6 ± 2.4	0.5681	−6.8 ± 2.8 (0.0166)	−6.4 ± 2.8 (0.0210)	−3.0 ± 2.7 (0.2677)	0.5657
HDL-C (mg/dL)	0.1 ± 0.8	0.7 ± 0.8	1.5 ± 0.9	0.4817	0.1 ± 1.1 (0.9185)	0.4 ± 1.1 (0.7153)	1.5 ± 1.1 (0.1863)	0.6688
non-HDL-C (mg/dL)	−11.2 ± 2.3	−9.6 ± 2.3	−6.7 ± 2.4	0.3786	−8.6 ± 3.1 (0.0059)	−10.0 ± 3.0 (0.0011)	−5.1 ± 3.0 (0.0886)	0.4947
VLDL-C (mg/dL)	−2.8 ± 1.1	−2.7 ± 1.1	−0.8 ± 1.1	0.3232	−2.6 ± 1.5 (0.0871)	−3.4 ± 1.5 (0.0236)	−2.1 ± 1.5 (0.1591)	0.8191
TG (mg/dL)	−4.9 ± 3.8	−5.5 ± 3.7	−4.1 ± 3.9	0.9622	−5.0 ± 5.5 (0.3603)	−10.5 ± 5.3 (0.0509)	−11.1 ± 5.3 (0.0371)	0.6853
VLDL-TG (mg/dL)	−1.3 ± 3.9	−1.2 ± 3.8	−4.1 ± 4.0	0.8343	−3.5 ± 5.7 (0.5446)	−6.6 ± 5.6 (0.2383)	−10.3 ± 5.5 (0.0645)	0.6951
Lp (a) (mg/dL)	−1.4 ± 1.0	−0.3 ± 1.0	0.7 ± 1.0	0.3405	−1.2 ± 1.3 (0.3669)	0.7 ± 1.3 (0.5798)	1.0 ± 1.3 (0.4590)	0.4471
apoB (mg/dL)	−8.3 ± 1.6	−6.5 ± 1.6	−4.8 ± 1.6	0.2939	−6.6 ± 2.1 (0.0019)	−7.1 ± 2.0 (0.0007)	−4.2 ± 2.0 (0.0392)	0.5654

Values are mean ± SEM. Change in parameters and *p*-value calculated with multivariate regression adjusting for age, gender, baseline parameter value, baseline BMI, treatment sequence, walnuts as snack or meal; *p*-value refers to differences between groups; values in brackets are *p*-values referring to the comparison walnut vs. control within the diet group. TC, total cholesterol; LDL-C, low density lipoprotein-cholesterol; HDL-C, high density lipoprotein-cholesterol; VLDL-C, very low density lipoprotein-cholesterol; TG, triglycerides; Lp (a), lipoprotein (a); apoB, apolipoproteinB.

**Table 4:** Effect of walnut consumption on fasting lipids, apoB, and Lp (a) when walnuts were consumed with meals or as snack.

	Walnuts				Difference between Walnuts and Control	
Parameter	Meal	Snack	*p*	Meal	Snack	*p*
TC (mg/dL)	−11.6 ± 2.2	−7.0 ± 2.3	0.1277	−7.3 ± 2.7 (0.0077)	−7.1 ± 2.8 (0.0126)	0.9515
LDL-C (mg/dL)	−8.3 ± 1.9	−6.0 ± 2.0	0.3948	−5.2 ± 2.2 (0.0222)	−5.7 ± 2.3 (0.0150)	0.8714
HDL-C (mg/dL)	0.6 ± 0.7	0.9 ± 0.7	0.7889	1.4 ± 0.9 (0.1058)	−0.2 ± 0.9 (0.8646)	0.212
non-HDL-C (mg/dL)	−11.3 ± 1.9	−7.0 ± 2.0	0.1039	−8.6 ± 2.4 (0.0005)	−7.1 ± 2.5 (0.0056)	0.6538
VLDL-C (mg/dL)	−3.2 ± 0.9	−1.0 ± 0.9	0.0648	−3.8 ± 1.2 (0.0017)	−1.5 ± 1.2 (0.2254)	0.1809
TG (mg/dL)	−7.5 ± 3.0	−2.1 ± 3.2	0.2057	−12.9 ± 4.3 (0.0029)	−4.7 ± 4.4 (0.2919)	0.182
VLDL-TG (mg/dL)	−4.5 ± 3.2	−0.1 ± 3.3	0.2964	−9.2 ± 4.5 (0.0427)	−4.4 ± 4.6 (0.3461)	0.4596
Lp (a) (mg/dL)	−0.3 ± 0.8	−0.3 ± 0.8	0.9745	0.4 ± 1.1 (0.7208)	−0.02 ± 1.1 (0.9841)	0.7927
apoB (mg/dL)	−8.6 ± 1.3	−4.5 ± 1.3	0.024	−6.5 ± 1.6 (0.0001)	−5.3 ± 1.7 (0.0022)	0.6024

Values are mean ±SEM. Change in parameters and *p*-values calculated with multivariate regression adjusting for age, gender, baseline parameter value, baseline BMI, treatment sequence, walnuts as snack or meal; *p*-values refer to differences between groups; values in brackets are *p*-values referring to the comparison of walnut-phase vs. control within the group. TC, total cholesterol; LDL-C, low density lipoprotein-cholesterol; HDL-C, high density lipoprotein-cholesterol; VLDL-C, very low density lipoprotein-cholesterol; TG, triglycerides; Lp (a), lipoprotein (a); apoB, apolipoproteinB.

## Supplementary Materials

The following are available online at http://www.mdpi.com/2072-6643/9/10/1097/s1, Table S1: Intention to treat analysis examining differences between treatment with walnuts and controls with all missing values imputed using single MCMC imputation; Table S2: Intention to treat analysis examining effect of walnut consumption on fasting lipids, apoB and Lp (a) when walnuts replace carbohydrates, fat or both with all missing values imputed using single MCMC imputation; Table S3: Effect of walnut consumption on fasting lipids, apoB and Lp (a) when walnuts were consumed with meals or as snack in an intention to treat analysis.

## Abbreviations

apoB	apolipoprotein B
BMI	body mass index
CH	carbohydrates
comb	combined
CVD	cardiovascular disease
ET-1	endothelin 1
F	fat
HbA1c	hemoglobin A1c
HDL-C	high-density lipoprotein cholesterol
hsCRP	high-sensitive C-reactive protein
LDL-C	low-density lipoprotein cholesterol
Lp (a)	lipoprotein a
n-3	omega-3
PUFA	polyunsaturated fatty acids
TG	triglycerides
TC	total cholesterol
VCAM-1	vascular cell adhesion molecule-1
VLDL-C	very-low-density lipoprotein cholesterol
