# Supplementary material for: A Walnut-Enriched Diet Reduces Lipids in Healthy Caucasian Subjects, Independent of Recommended Macronutrient Replacement and Time Point of Consumption: A Prospective, Randomized, Controlled Trial
Source: Nutrients. 2017 Oct 6;9(10):1097. doi: 10.3390/nu9101097 (PMC5691297; doi:10.3390/nu9101097)
Supplement: Supplementary file 1 [file nutrients-09-01097-s001.docx]

Supplemental Material:

**Table S1.** Intention to treat analysis examining differences between treatment with walnuts and controls with all missing values imputed using single Marcov chain Monte Carlo (MCMC) imputation.

| **Parameter** | **Baseline_W_** | **Post-value_w_** | **ΔWalnut** | **Baseline_C_** | **Post-value_c_** | **ΔControl** | **P value for change/post** |
| --- | --- | --- | --- | --- | --- | --- | --- |
| TC (mg/dl) | 231.5 ± 2.6 | 223.1 ± 1.6 | -8.6 | 231.7 ± 2.5 | 229.2 ± 1.6 | -2.4 | *0.0016/0.0016 |
| LDL-C (mg/dl) | 145.9 ± 2.2 | 139.3 ± 1.3 | -6.6 | 145.8 ± 2.1 | 143.6 ± 1.3 | -2.3 | *0.0074/0.0074 |
| HDL-C (mg/dl) | 68.5 ± 1.1 | 69.2 ± 0.5 | 0.6 | 68.7 ± 1.2 | 68.3 ± 0.5 | -0.3 | 0.1428/0.1428 |
| non-HDL-C (mg/dl) | 163.0 ± 2.4 | 154.4 ± 1.3 | -8.6 | 163.0 ± 2.3 | 161.5 ± 1.4 | -1.6 | *< .0001/< .0001 |
| VLDL-C (mg/dl) | 17.1 ± 1.0 | 15.0 ± 0.6 | -2.1 | 17.2 ± 1.1 | 18.0 ± 0.8 | 0.9 | *0.0005/0.0005 |
| TG (mg/dl) | 102.6 ± 3.3 | 98.1 ± 2.2 | -5.3 | 104.2 ± 3.4 | 107.9 ± 3.0 | 4.5 | *0.0016/0.0016 |
| VLDL-TG (mg/dl) | 75.4 ± 3.5 | 74.5 ± 2.3 | -2.8 | 79.2 ± 3.6 | 82.3 ± 3.1 | 5.0 | *0.0159/0.0159 |
| Lp(a) (mg/dl) | 12.5 (1-139) | 29.8 ± 0.6 | -0.4 | 12.0 (2 - 173) | 29.7 ± 0.5 | -0.5 | 0.8698/0.8698 |
| Log(Lp(a)) (mg/dl) | 2.5 (0 – 4.9) | 2.7 ± 0.02 | -0.02 | 2.5 (0.7 – 5.2) | 2.7 ± 0.02 | -0.01 | 0.7324/0.7324 |
| apoB (mg/dl) | 109.8 ± 1.5 | 103.4 ± 0.9 | -6.4 | 109.7 ± 1.4 | 108.8 ± 0.9 | -1.0 | *< .0001/< .0001 |
| HbA1c (mg/dl) | 5.4 ± 0.02 | 5.53 ± 0.01 | 0.07 | 5.5 ± 0.02 | 5.48 ± 0.01 | 0.03 | 0.0024/0.0024 |
| Glucose (mg/dl) | 91.3 ± 0.6 | 93.3 ± 0.5 | 1.4 | 92.5 ± 0.6 | 92.2 ± 0.5 | 0.3 | 0.0724/0.0724 |

Baseline values are mean ± SEM and median (range) for lipoprotein (a) and log of lipoprotein (a); Post-value and change in parameters are adjusted least square mean values, and together with the p-value, are calculated using a multivariate regression (proc mixed in SAS) adjusting for age, gender, baseline parameter, baseline body mass index (BMI), treatment sequence, type of diet reduction (fat, carbohydrate, or both), and whether walnuts were ingested as a snack or meal. There is not a difference in p-value based on whether the change or post value is used as a dependent variable. All values in bold print are significant.

**Table S2.** Intention to treat analysis examining the effect of walnut consumption on fasting lipids, apoB, and Lp(a) when walnuts replace carbohydrates, fat or both with all missing values imputed using single MCMC imputation.

|  | | **walnuts** | | | | **difference between walnuts and control** | | | |
| --- | --- | --- | --- | --- | --- | --- | --- | --- | --- |
| Parameter | Δcarbohydrate | | Δfat | Δcomb | p | Δcarbohydrate | Δfat | Δcomb | p |
| TC (mg/dl) | -11.1 ± 2.6 | | -8.5 ± 2.5 | -5.9 ± 2.7 | 0.3667 | -7.2 ± 3.4 (0.0344) | -7.7 ± 3.3 (0.0198) | -3.6 ± 3.4 (0.2911) | 0.6341 |
| LDL-C (mg/dl) | -8.2 ± 2.2 | | -6.4 ± 2.2 | -5.1 ± 2.4 | 0.6126 | -5.5 ± 2.8 (0.0455) | -4.7 ± 2.7 (0.0808) | -2.5 ± 2.8 (0.3597) | 0.7258 |
| HDL-C (mg/dl) | 0.1 ± 0.8 | | 1.0 ± 0.8 | 1.4 ± 0.8 | 0.4676 | 0.2 ± 1.1 (0.8199) | 1.0 ± 1.1 (0.3463) | 1.5 ± 1.1 (0.1703) | 0.7162 |
| non-HDL-C (mg/dl) | -10.4 ± 2.3 | | -8.8 ± 2.2 | -6.5 ± 2.4 | 0.4702 | -7.4 ± 3.0 (0.0157) | -9.1 ± 3.0 (0.0024) | -4.7 ± 3.0 (0.1237) | 0.5763 |
| VLDL-C (mg/dl) | -2.9 ± 1.0 | | -2.3 ± 1.0 | -1.0 ± 1.1 | 0.4245 | -2.8 ± 1.5 (0.0599) | -4.1 ± 1.4 (0.0053) | -2.1 ± 1.5 (0.1603) | 0.6192 |
| TG (mg/dl) | -4.7 ± 3.6 | | -5.2 ± 3.5 | -4.2 ± 3.8 | 0.9798 | -5.6 ± 5.3 (0.2998) | -12.6 ± 5.2 (0.0162) | -11.0 ± 5.3 (0.0408) | 0.6146 |
| VLDL-TG (mg/dl) | -1.8 ± 3.8 | | 0.5 ± 3.7 | -4.8 ± 4.0 | 0.6093 | -4.6 ± 5.6 (0.4129) | -8.1 ± 5.4 (0.1361) | -10.5 ± 5.5 (0.0592) | 0.7476 |
| Lp(a) (mg/dl) | -1.4 ± 1.0 | | -0.1 ± 1.0 | 0.5 ± 1.0 | 0.3422 | -1.3 ± 1.3 (0.3266) | 0.7 ± 1.3 (0.5774) | 0.9 ± 1.3 (0.4845) | 0.7058 |
| apoB (mg/dl) | -8.0 ± 1.5 | | -6.1 ± 1.5 | -4.8 ± 1.6 | 0.3225 | -6.0 ± 2.0 (0.0033) | -6.2 ± 2.0 (0.0019) | -4.0 ± 2.0 (0.0483) | 0.6935 |

Walnut columns shows the least square mean change in laboratory parameters according to the type of reduction in diet (fat, carbohydrate, or both) in the walnut treatment phase, determined with a multivariate regression model (proc mixed in SAS). Values adjusted for: age, gender, baseline parameter, baseline BMI, treatment sequence, and whether walnuts were ingested as a snack or meal.

Difference between walnut and control columns shows the least square mean difference in change in laboratory parameters between the walnut and control phase according to type of reduction in diet (fat, carbohydrate, or both) determined with a multivariate regression model (proc mixed in SAS), and an interaction term between treatment and type of reduction in diet (fat, carbohydrate, or both). Values adjusted for: age, gender, baseline parameter, baseline BMI, treatment sequence, and whether walnuts were ingested as a snack or meal.

**Table S3.** Effect of walnut consumption on fasting lipids, apoB, and Lp(a) when walnuts were consumed with meals or as snack in an intention to treat analysis.

|  |  | **walnuts** |  |  | **difference between walnuts and control** |  |
| --- | --- | --- | --- | --- | --- | --- |
| Parameter | meal | snack | p | meal | snack | *p* |
| TC (mg/dl) | -11.5 ± 2.2 | -5.6 ± 2.2 | 0.0433 | -7.0 ± 2.7 (0.0102) | -5.3 ± 2.7 (0.0548) | 0.6519 |
| LDL-C (mg/dl) | -8.1 ± 1.9 | -5.1 ± 1.9 | 0.2410 | -4.8 ± 2.2 (0.0317) | -3.7 ± 2.2 (0.0983) | 0.7325 |
| HDL-C (mg/dl) | 0.6 ± 0.7 | 1.1 ± 0.7 | 0.5742 | 1.6 ± 0.9 (0.0775) | 0.3 ± 0.9 (0.7618) | 0.3027 |
| non-HDL-C (mg/dl) | -11.2 ± 1.9 | -5.9 ± 1.9 | 0.0371 | -4.8 ± 2.2 (0.0317) | -3.7 ± 2.2 (0.0983) | 0.4165 |
| VLDL-C (mg/dl) | -3.3 ± 0.9 | -0.8 ± 0.9 | 0.0307 | -3.9 ± 1.2 (0.0013) | -2.1 ± 1.2 (0.0826) | 0.2916 |
| TG (mg/dl) | -8.2 ± 3.0 | -1.2 ± 3.0 | 0.0891 | -13.6 ± 4.3 (0.0018) | -5.9 ± 4.3 (0.1743) | 0.2097 |
| VLDL-TG (mg/dl) | -4.7 ± 3.1 | -0.6 ± 3.2 | 0.2134 | -9.6 ± 4.5 (0.0322) | -5.8 ± 4.5 (0.2001) | 0.5457 |
| Lp(a) (mg/dl) | -0.2 ± 0.8 | -0.5 ± 0.8 | 0.8250 | 0.5 ± 1.0 (0.6457) | -0.2 ± 1.0 (0.8171) | 0.6257 |
| apoB (mg/dl) | -8.6 ± 1.3 | -4.1 ± 1.3 | *0.0101 | -6.2 ± 1.6 (0.0002) | -4.5 ± 1.6 (0.0060) | 0.4623 |

Walnut columns show the least square mean change in laboratory parameters, according to whether walnuts were ingested with meals or as a snack in the walnut treatment phase, determined with a multivariate regression model (proc mixed in SAS). Values adjusted for: age, gender, baseline parameter, baseline BMI, treatment sequence, and type of diet reduction (fat, carbohydrate, or both). The difference between walnut and control columns shows the least square mean difference in change in laboratory parameters between the walnut and control phase, according to whether walnuts were ingested with meals or as a snack, which was determined with a multivariate regression model (proc mixed in SAS), and an interaction term between treatment and whether walnuts were ingested as a snack or meal. Values adjusted for: age, gender, baseline parameter, baseline BMI, treatment sequence, and type of reduction in diet (fat, carbohydrate, or both). Single imputation of missing values was performed using a Marcov chain Monte Carlo (MCMC) method (using information available on age, gender, BMI, treatment phase, and other laboratory values considered).
